# Biosafety and efficacy of Kv7 activating rdHSV-CA8∗ analgesic gene therapy for chronic pain via the intra-articular route in mice

**DOI:** 10.1016/j.ymthe.2025.05.029

**Published:** 2025-05-28

**Authors:** Roy C. Levitt, Munal B. Kandel, Gerald Z. Zhuang, William F. Goins, Konstantinos D. Sarantopoulos, Joseph C. Glorioso

**Affiliations:** 1 Department of Anesthesiology, Perioperative Medicine and Pain Management, University of Miami Miller School of Medicine, Miami, FL, USA; 2 Bascom Palmer Eye Institute, University of Miami Miller School of Medicine, Miami, FL, USA; 3 John T. MacDonald Foundation Department of Human Genetics, University of Miami Miller School of Medicine, Miami, FL, USA; 4 John P. Hussman Institute for Human Genomics, University of Miami Miller School of Medicine, Miami, FL, USA; 5 Department of Microbiology and Molecular Genetics, University of Pittsburgh School of Medicine, Pittsburgh, PA, USA

**Keywords:** carbonic anhydrase-8, allodynia, hyperalgesia, osteoarthritis, Kv7 voltage-gated potassium channels, cytosolic free calcium, ITPR1, replication defective Herpes-1 virus, gene therapy, chronic pain

## Abstract

Chronic pain remains a global health challenge, often resistant to available treatments with socioeconomic and psychological burdens. All chronic pain is believed due to neuronal signaling imbalances, resulting in increased excitability. Gene therapy represents a promising molecular therapy targeting molecular pain processing pathways, by offering precise, localized, long-lasting neuromodulation while minimizing systemic exposure and side effects. In model systems, replication-defective, disease-free, herpes simplex virus (rdHSV) gene therapy expressing an analgesic carbonic anhydrase-8 (CA8∗) peptide variant corrects somatosensory hyperexcitability by activating Kv7 voltage-gated potassium channels, produces profound, long-lasting analgesia and treats chronic pain from knee osteoarthritis (OA). In these studies, we provide the first non-glucagon-like peptide (GLP) biosafety, efficacy, biodistribution, shedding, and histopathology examination of this rdHSV-CA8∗. Naive mice were examined for clinical safety, biodistribution across all major tissues, knee histopathology, and analgesic efficacy via the intra-articular knee route of administration. We observed no signs of persistent toxicity, viral genomes remained where they were injected, and there was no evidence of shedding. Profound analgesia persisted for 6 months without functional impairments. These initial biosafety and efficacy data support further development of rdHSV-CA8∗ for treating chronic knee pain due to moderate to severe OA.

## Graphical abstract

**Figure:**
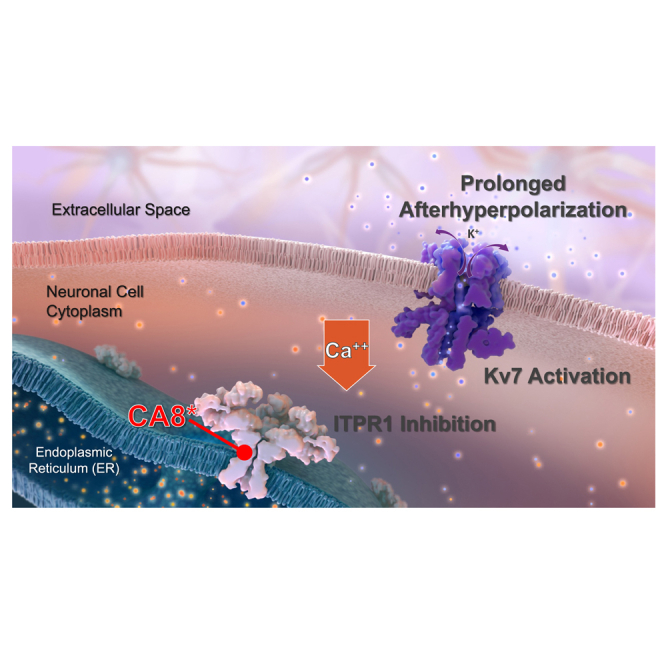

## Teaser

This report provides important non-GLP safety, biodistribution, shedding, histopathology, and efficacy data of rdHSV-CA8∗ gene therapy via the intra-articular knee route of administration. A single rdHSV-CA8∗ dose displayed no dose-limiting toxicity while demonstrating profound persistent analgesia for >6 months. rdHSV-CA8∗ may potentially transform the chronic pain treatment landscape with targeted neuromodulating molecular therapy.

## Introduction

Persistent pain, like many other serious neurological disorders, occurs when imbalances in excitatory and inhibitory signaling between neurons develop.^1^^,^^2^^,^^3^^,^^4^^,^^5^^,^^6^^,^^7^^,^^8^ Most of these illnesses represent serious undertreated global health challenges, often resistant to conventional therapeutics and associated with significant socioeconomic and psychological burdens. Pain is a protective mechanism that alerts the body to actual or potential harm. When pain persists beyond a period of usefulness (chronic pain), it represents a maladaptive response of the nervous system and a serious neurological disorder.^9^ Chronic pain as a neurological disorder impacts approximately 20% of the worldwide population, negatively affecting quality of life, morbidity, and mortality. Efforts to routinely better assess and manage pain in the clinic led to the adoption of pain as the fifth vital sign in 1996 by the American Pain Society.^10^ Despite advances in pain monitoring and management over the last 30 years, current therapeutic approaches are most often inadequate. A major limitation of most current chronic pain therapeutics is that they are systemic and interact with receptors and/or channels widely expressed throughout the peripheral and central nervous system (CNS). These so-called off-target effects can occur when the biochemical activity of a systemic drug acts other than on its intended target and produces adverse events that can limit its utility. There are many other variables, which may have negatively impacted the utility of chronic pain treatments and potentially viable new targets. For example, intolerable side effects of the most selective therapeutic interventions can occur due to the redundancy of the nervous system.^11^ For example, a relatively limited number of signaling molecules, including receptors, ion channels and second messenger systems, are found in a variety of organ systems producing cardiac, respiratory, hepatic, or CNS side effects. Still other factors such as age, sex, and genetic background can influence individual responses.

An absence of safe and effective treatment options for chronic pain over the last three decades along with increased monitoring and awareness of its prevalence, likely contributed to the evolution of the opioid epidemic. The use of opioid analgesics for chronic pain treatment is problematic because of life-threatening side effects, as well as physiological dependence, tolerance, abuse, diversion, and addiction. The limited efficacy of current therapeutics and their associated complications emphasizes the importance of developing innovative chronic pain therapeutic options, including the introduction of localized long-lived treatment with gene transfer or gene therapy strategies.^12^^,^^13^

Gene therapy represents a cutting-edge therapeutic strategy that delivers specific genetic material selectively to cells or tissues to express therapeutic molecules with various cellular effects. These biological molecules can modulate target cellular gene expression, express exogenous genes and gene products with potential therapeutic benefits, or correct genetic mutations that underlie rare diseases. This approach offers a unique capability to target the underlying maladaptive molecular and cellular biology underlying disease mechanisms, potentially providing long-lasting relief and minimal off-target effects associated with systemic exposure of conventional therapeutics that can lead to serious side effects.^14^^,^^15^ Over the past two decades, preclinical and early clinical studies have demonstrated the feasibility of using gene transfer to manage various pain syndromes by targeting pain signaling pathways, inflammatory mediators, and neural imbalances.^12^^,^^13^ Progress in gene therapies for chronic pain is reviewed elsewhere, describing advances in viral vectors such as adenovirus, helper-dependent adenovirus, adeno-associated virus (AAV), and retroviruses like lentivirus.^12^^,^^13^ We have previously conducted extensive proof of concept studies of a replication-defective, disease-free, herpes simplex virus (rdHSV) gene therapy expressing a novel analgesic carbonic anhydrase-8∗ (CA8∗) peptide variant treating the complex pain produced by moderate-to-severe knee joint (KJ) osteoarthritis (OA).^14^^,^^16^^,^^17^^,^^18^^,^^19^^,^^20^^,^^21^ In this study, we summarize the recent preclinical development of this novel chronic pain therapeutic incorporating this unique delivery system, including initial rdHSV-CA8∗ clinical safety, biodistribution, shedding, histopathology, and efficacy data of this promising new molecular therapy.

## Results

### Non-glucagon-like peptide preclinical safety and efficacy studies on vHCA8∗ gene therapy

A major limitation of AAV strains used in our prior studies was their limited potential to transduce DRG neurons except after direct intra-neural injections.^21^^,^^22^^,^^23^ In contrast, rdHSV-based gene therapy was able to transduce DRG neurons from a variety of routes of administration, including the sciatic nerve, rear footpad, and after intra-articular (IA) KJ injections,^14^^,^^24^^,^^25^^,^^26^ commonly used clinically for short-term localized treatment of chronic OA KJ pain. The direct IA KJ route of administration is appealing because it is expected to greatly limit peripheral nervous system (PNS) exposure, immune responses, and potential toxicity related to gene therapy, as compared with systemic or intra-thecal exposure. Previously, we were able to demonstrate that IA KJ injections of vHCA8∗ were able to infect synovial KJ primary afferents and treat moderate to severe OA knee pain in mice.^14^ One major advantage of these replication-defective viral vectors is that they provide no viral antigen targets for immune effector cells, and only the therapeutic gene product is expressed once the viral DNA genome is resident within the neuronal cell body. Consequently, these rdHSV vector systems are much less likely to produce exaggerated immune responses observed with other gene therapy vectors.^27^^,^^28^^,^^29^

### Analgesic dose-response, minimum effective analgesic dose, and preliminary non-glucagon-like peptide no observable adverse event level

If our analgesic was acting like a traditional local anesthetic, all sensory and motor functions would become markedly reduced or undetectable. Therefore, complementary pain assays were used to assess analgesia and preservation of sensory and motor functions (as described previously). Briefly, mechanical responses using calibrated von Frey fibers, thermal responses using Hargreave’s method,^30^ and motor functions using weight bearing.^14^ All in-life measures were made by individuals masked to treatment groups. Animals were randomly assigned to groups. Assays were conducted at approximately the same time of day throughout each experiment. Statistical methods were conducted as described previously. The preliminary non-glucagon-like peptide (GLP) no observable adverse event level (NOAEL) was determined using these clinical safety observational assessments.

### Clinical safety assessments in mice

Routine clinical safety assessments are made at Baseline and weekly thereafter for 8 weeks (e.g., general appearance, body weight, body temperature, daily cage checks for clinical safety assessments included abnormal grooming, vocalization, and general behavior [socialization]). There were no changes in body weight, body temperature, or any abnormal clinical safety observations during daily cage checks throughout the experiment in any treatment group (please see Figures S1 and S2) for body weight or body temperature results. Based on these clinical safety data in mice, vHCA8∗WT at the highest dose tested exhibits a desirable safety profile.

**Figure 1:**
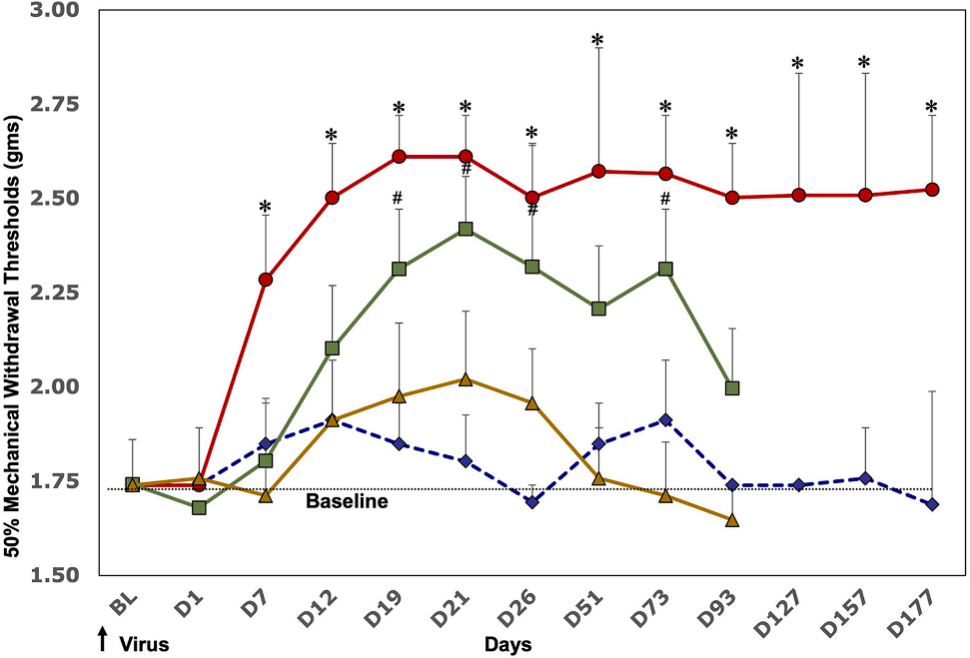
Mechanical withdrawal dose-responses, MED, in naive BALB/c mice Responses were evaluated for vHCA8∗WT after IA KJ injection at the HD (1E6 PFU) (red circles), MD (1E5 PFU) (green boxes), and low-dose (LD) (1E4 PFU) (gold triangles) in 6 μL. Mechanical thresholds in the vHCA8∗WT HD and MD doses were significantly higher than that observed for vHCA8∗MT HD negative controls (1E6 PFU) (blue diamonds, dashed line), as shown on various days. vHCA8∗WT at the LD failed to differ from vHCA8∗MT at HD through D154. Data are presented as average ± SEM, (*N* = 9) (*p* < 0.05 was considered significant; Student’s *t* test, one-way and repeated measure two-way ANOVA, ∗*p* < 0.05 for vHCA8∗WT-HD and #*p* < 0.05 for vHCA8∗WT-MD. Repeated measured two-way ANOVA results: (1) 13 time points for 4 groups of MT-HD, WT-HD, WT-MD, and WT-LD from BL to D92, significant differences in mechanical threshold were found between groups, F(3,32) = 37.25, *p* = 1.49E−10, and across all time points, F(17,544) = 4.05, *p* = 1.15E−07. There were no significant interactions between time points and groups [F(51,544) = 1.02, *p* = 0.43]. (2) Twenty-one time points for 2 groups of MT-HD and WT-HD from BL-D177, significant differences in mechanical threshold were found between groups, F(1,16) = 208.54, *p* = 37.25, *p* = 1.35E−10, and across all time points, F(20,320) = 2.06, *p* = 5.3E−03. There were no significant interactions between time points and groups, F(20,320) = 1.39, *p* = 0.12.

Dose-ranging was conducted using 10-fold dilutions of virus to determine the minimum effective analgesic dose (MED) by the route selected (IA KJ injections). Figure 1 shows the mechanical withdrawal dose-response curves obtained from IA KJ injection of the vHCA8∗WT in naive male BALB/c mice. Mechanical responses after vHCA8∗WT IA KJ injection at the highest dose (1E6 PFU) were increased above baseline and significantly greater than the vHCA8∗MT (1E6 PFU) negative control starting on D7 and persisting through D177. Additionally, we found that mechanical thresholds in mice treated with vHCA8∗WT at 1E5 PFU (mid dose [MD]) were significantly greater than the vHCA8∗MT negative control on D19–26 and 73, and consistently above baseline during that time frame. Mechanical thresholds in mice treated with the lowest vHCA8∗WT (1E4 PFU) dose were not increased above baseline or significantly different than vHCA8∗MT (1E6 PFU) high dose (HD). These data also provide evidence of a durable analgesic response (out to ∼6 months) after KJ injection of vHCA8∗WT at the highest dose (1E6 PFU). We also tested whether thermal sensory function was preserved during the same experiment.

**Figure 2:**
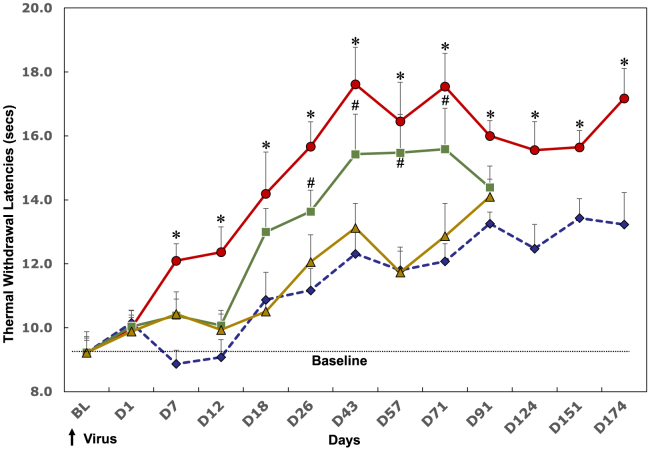
Thermal withdrawal dose responses (latencies in seconds), MED, in naive BALB/c mice Thermal withdrawal latencies were evaluated for vHCA8∗WT after IA KJ injection of HD (1E6 PFU) (red circles), MD (1E5 PFU) (green boxes), and low-dose (LD) (1E4 PFU) (gold triangles) in 6 μL. Thermal withdrawal latencies for the vHCA8∗WT HD and MD were significantly higher than that observed for vHCA8∗MT HD (1E6 PFU) (blue diamonds, dashed line) negative controls, as shown on various days out to 6 months. Thermal responses in mice treated with the lowest vHCA8∗WT dose (1E4 PFU) did not differ from mice treated with vHCA8∗MT HD negative control through D91. At no time did any treatment group reach the maximum withdrawal latency cut-off of 25 s. These data are consistent with preservation of thermal withdrawal responses. Data are presented as average ± SEM (*N* = 9) (*p* < 0.05 was considered significant; Student t-test, one-way and two-way repeated measure ANOVA), ∗*p* < 0.05 for vHCA8∗WT-HD, #*p* < 0.05 vHCA8∗WT-MD. Figure 2 repeated measured two-way ANOVA results: (1) 13 time points for 4 groups of MT, WT-HD, WT-MD and WT-LD from BL to D91, Significant differences in mechanical threshold were found between groups, F(3,32) = 57.54, *p* = 5.46E−13, across all time points, F(17,544) = 7.82, *p* = 1.25E−17. There were no significant interactions between time points and groups, F(51,544) = 1.17, *p* = 0.2. (2) Twenty-one time points for 2 groups of MT and WT-HD from BL-D174, significant differences in mechanical threshold were found between groups, F(1,16) = 281.83, *p* = 1.4E−11, across all time points, F(20,320) = 4.05, *p* = 3.83E−8. There was also significant difference in interactions between time points and groups, F(20,320) = 2.08, *p* = 4.72E−03.

Figure 2 shows that thermal withdrawal latencies for the vHCA8∗WT vector at the HD (1E6 PFU) were preserved through D174 using the Hargreaves’s method (Ugo Basile Thermal Plantar, Stoelting Co.).^30^ At no time did any treatment group reach the maximum thermal withdrawal latency cut-off of 25 s, indicating that thermal sensory responses are preserved despite the increase above baseline for the HD and MD. Thermal withdrawal latencies for mice treated with vHCA8∗WT-HD and -MD were significantly higher than that observed for vHCA8∗MT HD negative controls, as shown at various days out to D174 in Figure 2. Once again, there was a sustained analgesic response from D7 through D174 in mice treated with IA KJ injections of vHCA8∗WT HD. vHCA8∗WT MD results are consistent with a MED of 1E5 PFU.

**Figure 3:**
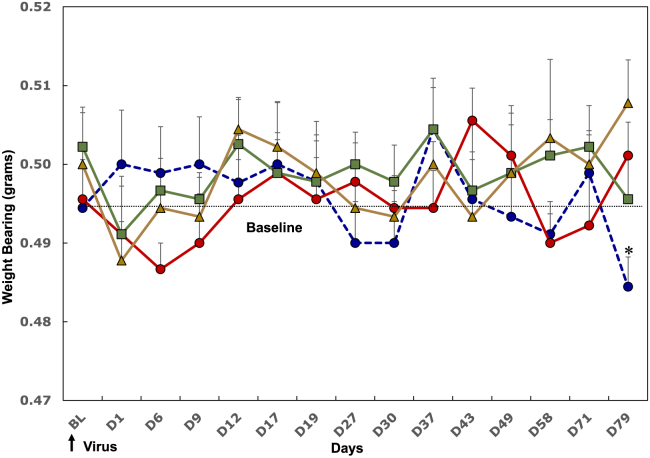
Weight-bearing (g) responses are preserved in naive BALB/c mice Weight bearing as evaluated for vHCA8∗WT after IA KJ injection of HD (1E6 PFU) (red circles), MD (1E5 PFU) (green boxes), and low-dose (LD) (1E4 PFU) (gold triangles) in 6 μL. Data are presented as average ± SEM, *N* = 9, ∗*p* < 0.05 significant. There were no significant differences in weight-bearing between CA8∗MT-HD control (1E6 PFU) (blue diamonds, blue dashed line) and CA8∗WT-HD, -MD or -LD from BL to D71. D79 CA8∗MT HD differed from CA8∗HD, CA8∗MD and CA8∗LD. Data are presented as ratio of left (ipsilateral − treated)/left (ipsilateral − treated) + right (contralateral − untreated); or left/(left + right). One-way ANOVA Fisher’s LSD *post hoc* test was used to analyze difference between groups at each time point.

We also tested whether weight bearing as a measure of motor functions was preserved during this experiment in naive BALB/c mice. Figure 3 shows that weight-bearing was unchanged through D71 for all treatment groups. vHCA8∗MT differed from vHCA8∗WT groups only on D79. These data demonstrate motor functions were preserved after vector treatments in all groups.

Overall, these results in naive BALB/c mice, including clinical safety observations, are consistent with an acceptable safety profile and we concluded that 1E6 PFU is the preliminary non-GLP NOAEL based on clinical safety observations; that the MED is 1E5 PFU, and analgesia durability was shown to be at least 6 months. We also demonstrate the preservation of sensory and motor functions (Figures 1–3) over a 6-month time frame. These data also demonstrate that IA KJ injections of vHCA8∗WT HD or MD does not act like a traditional local anesthetic that produces complete loss of all sensory and motor functions, despite evidence of significant long-lasting analgesia (Figures 1 and 2).

### Biodistribution and shedding experiments

We had previously selected the IA knee route of administration for vHCA8∗ given its clinical ease and general use for administering current knee OA treatments.^14^ Initial non-GLP biodistribution was conducted with this route using qPCR to detect viral DNA encoding for *glycoprotein D* (*gD*) gene, encoding for a major structural component of the HSV envelope essential to viral entry into cells. Biodistribution results show a viral DNA signal (qPCR for *gD*) in three tissues (i.e., KJ, adjacent quadriceps muscle, and bone marrow contiguous with the KJ) (Table 1, top, middle, and bottom, respectively). This signal was strongest in the KJ and weakest (nearly background) in muscle and bone marrow specimens on D1 and afterward. The KJ signal decreased through D28 and was at background levels on D14 and D28 for muscle and bone marrow. Additionally, there was no *gD* qPCR signal in the heart, liver, spleen, kidney, testicle, brain, or spinal cord (dorsal horn, ventral horn) consistent with very restricted vector biodistribution, limited only to local tissues. We were unable to detect a shedding signal using *gD* qPCR (lower limits of quantification of ∼50 copies/1,000 ng DNA) in the salivary gland, urine, and blood after KJ administration of vHCA8∗WT at the HD (1E6 PFU). Based on these data demonstrating localized biodistribution, further microscopic safety histology analyses were focused on only those tissues that demonstrated a *gD* qPCR signal (KJ, quadriceps muscle, and bone marrow) (Kristina Ullrich, PhD, personal communication).

**Table 1:** Summary of non-GLP biodistribution and shedding results using qPCR (*gD*)

**Biodistribution (KJ) (copies/1,000 ng gDNA)**							
**Animal number**		**1**	**2**	**3**	**4**	**5**	**Mean**
Day 1	HD	747,413	524,351	91,458	269,291	267,727	380,048
	MD	31,812	4,801	17,924	28,707	–	20,811
Day 14	HD	1,548	1,517	1,580	–	–	1,548
	MD	1,303	489	2,363	1,476	–	1,408
Day 28	HD	1,168	1,133	1,644	2,013	1,677	1,527

MD	572	860	1,193	1,034	–	915
**Biodistribution (quadriceps muscle, adjacent to KJ) (copies/1,000 ng gDNA)**							

**Mouse**		**1**	**2**	**3**	**4**	**5**	**Mean**

Day 1	HD	1,001	853	767	5,467	–	2,022
	MD	0	0	514	0	–	129
Day 14	HD	0	0	0	0	0	0
	MD	0	0	0	790	–	198
Day 28	HD	0	0	0	366	0	73

MD	0	0	0	0	–	0
**Biodistribution (bone marrow, femur) (copies/1,000 ng gDNA)**							

**Mouse**		**1**	**2**	**3**	**4**	**5**	**Mean**

Day 1	HD	110	224	1,113	2,536	306	858
	MD	70	151	6	–	–	76
Day 14	HD	0	0	0	0	–	0
	MD	0	0	0	0	0	0
Day 28	HD	0	0	4	0	–	1
	MD	0	0	0	–	–	0

qPCR was run essentially as described elsewhere.^29^ Tissues were collected and genomic DNA extracted from male naïve BALB/c mice tissues after IA KJ injection with vHCA8∗WT at 1E6 PFU (HD), or 1E5 PFU (MD). Results shown are copies/100 ng of genomic DNA. Table 1 (top) includes results from KJ synovial tissues. Table 1 (middle) includes results from quadriceps muscle dissected from the region contiguous with each KJ. Table 1 (bottom) includes results from bone marrow derived from the femur adjacent to the KJ in each animal. *N* = 4–5 animals per group at D1, D14, or D28. (LLOQ ∼50 copies/1,000 ng DNA).

### Non-GLP microscopic histopathology of selected tissues

Left knees of naive BALB/c mice that underwent IA KJ injections with vHCA8∗WT or MT viral particles (1E6 PFU) in 6 μL containing 10% glycerol in dPBS without Ca^2+^ and Mg^++^) or the right knees were injected with vehicle controls (PBS) (*n* = 5 each, total 10 mice). At D28, animals were sacrificed using humane protocols approved by the University of Miami Miller School of Medicine Institutional Animal Care and Use Committee and animals were perfused with saline and 4% paraformaldehyde. KJs were dissected and after at least 12 h of fixation, tissues were decalcified for 30–36 h on a shaker platform at room temperature.

**Figure 4:**
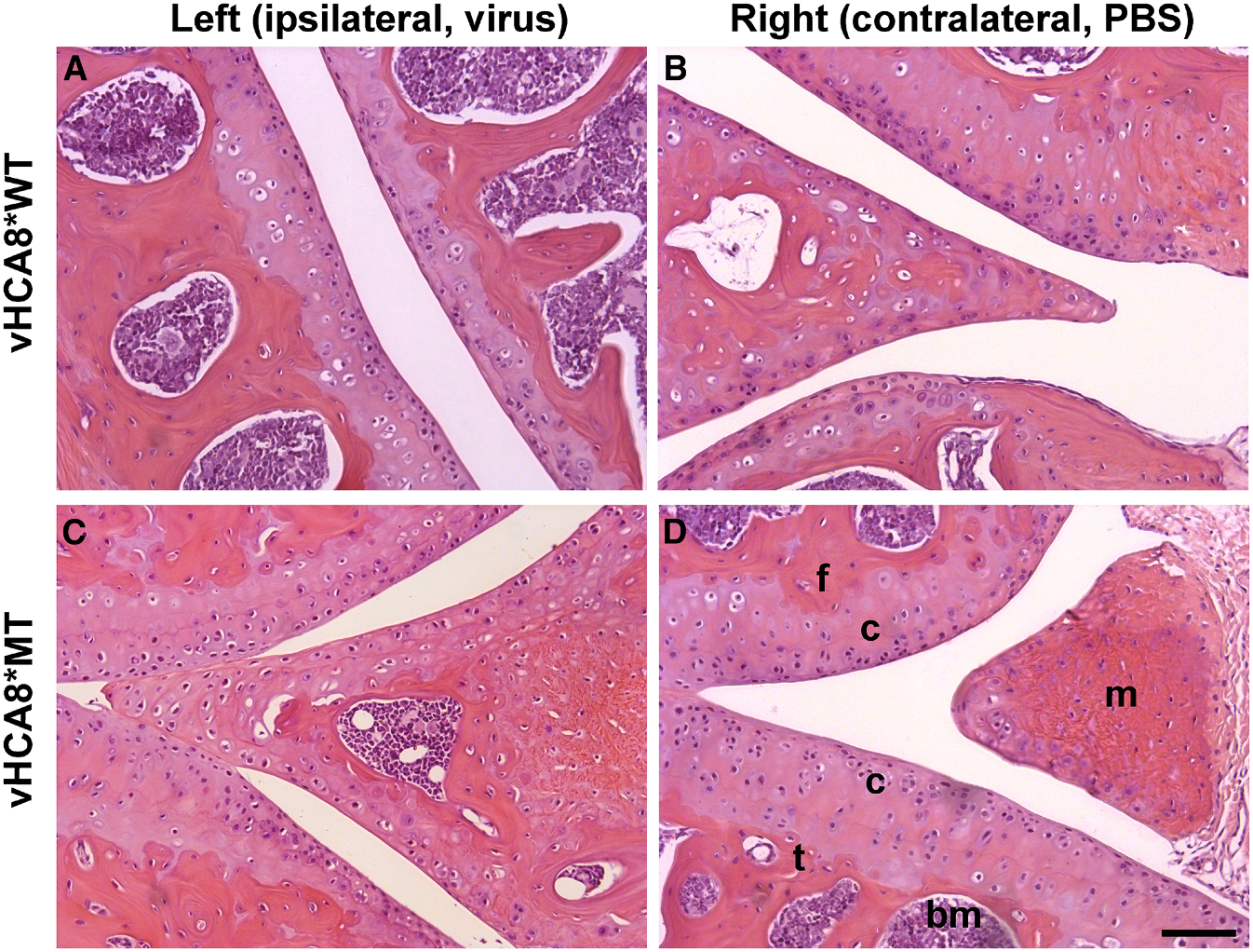
Initial non-GLP microscopic histological examination of KJ cartilage after IA KJ injections of vHCA8∗WT, vHCA8∗MT, and PBS (vehicle) KJ from naive BALB/c mice injected with (A) vHCA8∗WT HD (1E6 PFU), (B) PBS, or (C) vHCA8∗MT HD (1E6 PFU), and (D) PBS, harvested at D28. There were no adverse effects on KJ cartilage (c), meniscus (m), and bone marrow (bm) at D28 in vHCA8WT and MT groups, as compared with PBS and untreated (not shown) negative controls. f, femur; t, tibia. Scale bar, 100 μm. Results provided by a board-certified veterinary pathologist, Greenfield Pathology (independent CRO, Greenfield, IN). *N* = 5; scale bar, 100 μm.

**Figure 5:**
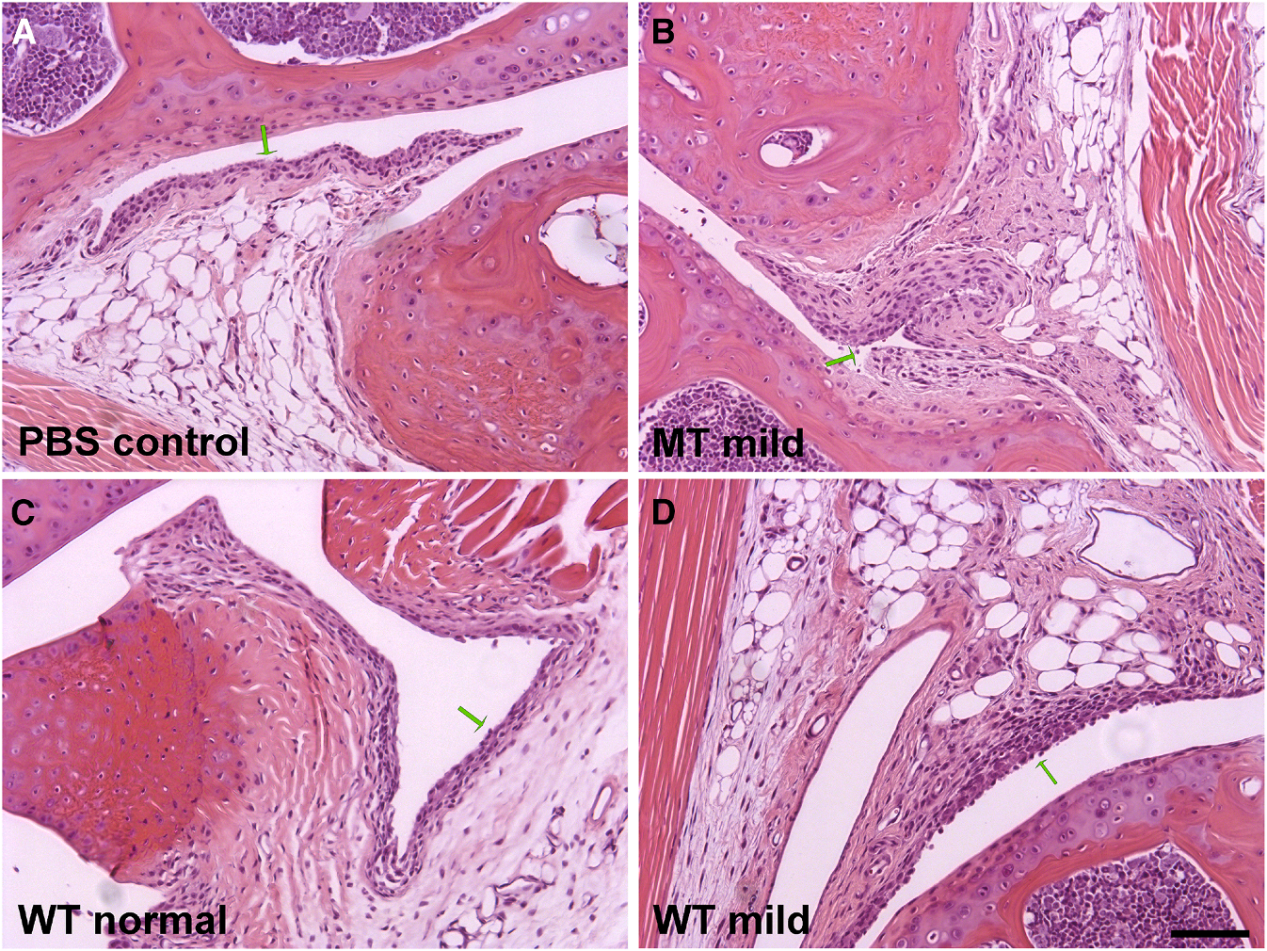
Microscopic histological examination shows synovium minimal to mild transient reaction after IA KJ injections of vHCA8∗ and controls KJ from naive BALB/c mice injected with (A) PBS negative control, (B) vHCA8∗MT HD (1E6 PFU), and (C and D) vHCA8∗WT HD (1E6 PFU) at D28. vHCA8∗WT and MT, but not PBS produced minimal to mild reactions in synovium area after injection into KJs of naive animals. *N* = 5; scale bar, 100 μm.

Tissues were delivered to University of Miami Department of Pathology for embedding, sectioning, hematoxylin and eosin staining and sent to Greenfield Pathology Services, Inc (Greenfield, IN) for evaluation by a board-certified pathologist. Microscopic analyses of the KJ at different time points are ongoing. KJ cartilage and structure overall were unchanged through D28 (Figure 4) after IA KJ injection of vHCA8∗WT at the highest dose in vHCA8∗ (ipsilateral KJ) and vehicle-treated controls (contralateral KJ). All bone marrow sections were read as normal compared with PBS-injected control animals. Additional microscopic analyses showed minimal-to-mild synovial membrane inflammation in both vHCA8∗WT and vHCA8∗MT treatments, but not in PBS controls. These data suggest a low-grade viral inflammatory response or injection site reaction (ISR) at D14 (Figure 5) (Greenfield pathology). Synovial membranes seem to be less inflamed at D28 compared with D14 with complete resolution by D90 and D180 (data not shown).

From these data we conclude that 1E6 PFU is the non-GLP NOAEL, 1E5 PFU is the MED, and analgesia durability was shown to be at least 6 months. There was no evidence of traditional local anesthetic effects with the preservation of sensory functions through 6 months. Additionally, there was no evidence for loss of motor functions in any groups. Biodistribution was demonstrated to be very limited and confined to the KJ and adjacent bone marrow and muscle. Finally, there was no evidence of clinical toxicity or durable microscopic histopathology consistent with a preliminary non-GLP NOAEL of 1E6 PFU. The was no evidence of dose-limiting toxicity in these studies. Mice treated with the highest dose of vHCA8∗WT (1E6 PFU) demonstrated profound persistent analgesia without decrement out to ∼6 months. The only safety finding observed was microscopic histopathology representing a transient ISR that was minimal to mild identified in both vHCA8∗WT and vHCA8∗MT vector-treated animals.

### Estimating morphine milligram equivalents from murine analgesia measurements

**Figure 6:**
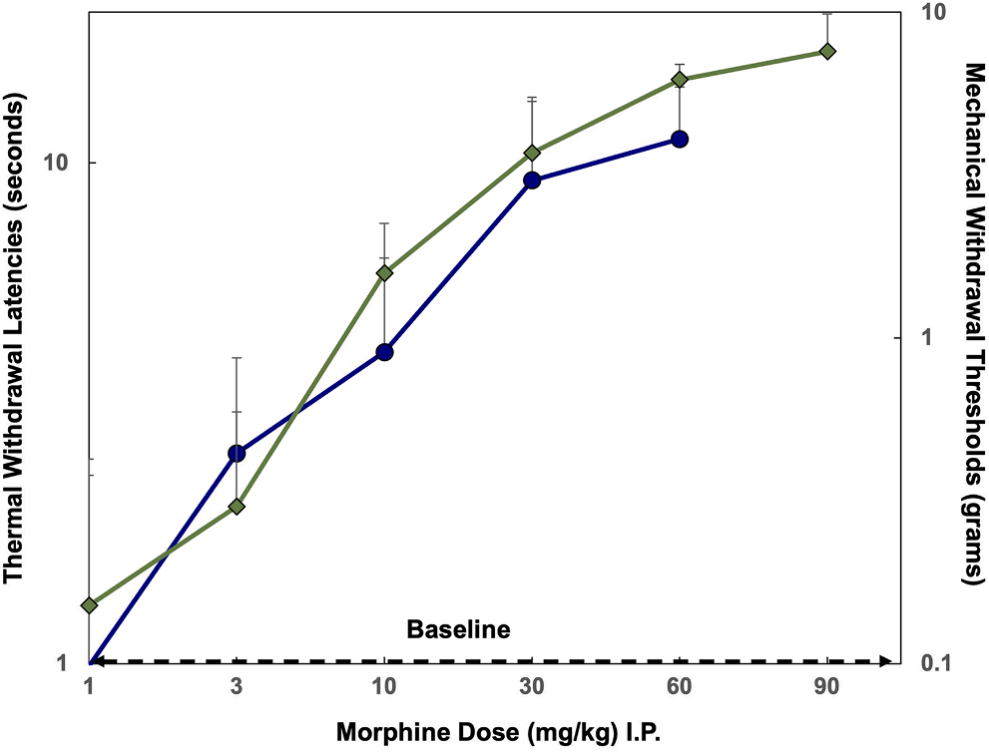
Estimating morphine milligram equivalents in humans from murine analgesia measurements Eight- to 10-week-old naive male C57BL/6J mice were injected i.p. with morphine at each dose diluted in saline. Saline was used as vehicle control and all results were normalized to saline response. Hargreaves method was used to measure thermal responses, as described previously.^16^^,^^20^ Mechanical responses were measured 30–60 min after i.p. administration of morphine, like that described previously.^16^^,^^20^ The left axis (log10) denotes mean ± SEM thermal withdrawal latencies (seconds) at each dose of i.p. morphine. The right axis (log10) denotes mean ± SEM mechanical withdrawal response (grams) at each dose of i.p. morphine. (*N* = 8); ∗∗*p* < 0.0001, ∗*p* < 0.05 vs. saline controls. Thermal data are reported previously by Fu et al. 2017.^16^

We observed increased mechanical withdrawal responses of ∼0.7–0.8 g and 3–4 s in thermal withdrawal responses over ∼6 months after a single treatment in mice with vHCA8∗ gene therapy (Figures 3 and 4). To translate these findings into analgesic equivalents, we carried out similar experiments in 8- to 10-week-old naive male C57BL/6J mice that were injected intraperitoneally (i.p.) with increasing doses of morphine diluted in saline. Saline was used as vehicle control and all results were normalized to saline response. Mechanical responses were measured using von Frey and the Hargreaves method was used to measure thermal responses, essentially as described previously.^20^ Each bar denotes mean ± SD (Adapted, in part from Fu et al., 2017^16^). We translated morphine analgesic responses from mice to humans based on morphine dose-response data using mechanical and thermal nociception (Figures 1, 2, and 6), and as reported previously only for thermal nociception.^16^ The amount of morphine administered in mice to produce antinociception (increase in mechanical and thermal responses above baseline) is known as the mouse analgesic equivalent dose. By obtaining mouse parenteral morphine analgesic equivalents, we can translate to a human oral morphine analgesic equivalent doses by allometric conversion, which depends on a formula that accounts for body surface area coefficients in humans and mice.^31^ The human oral dose (3× the parenteral equivalent dose) (mg/kg) equals the mouse morphine dose (mg/kg) (i.p.) × mouse Km^3^/human Km^37^.

After vHCA8∗ treatment we observed sustained increases of ∼5–6 s over baseline for thermal withdrawal latencies and ∼0.7–0.8 gm increases above baseline in mechanical withdrawal responses (Figures 1 and 2). Using this approach, these analgesia data translate to >85 mg of oral morphine based on thermal pain and, >100 mg of oral morphine based on mechanical pain assessments in an average sized (70 kg) adult dosed routinely throughout each day to maintain analgesia for ∼6 months (Figure 6).

## Discussion

### The role of peripheral mechanisms in the development and maintenance of chronic pain

Considerable preclinical and clinical evidence now indicates that CNS and PNS hypersensitivity (peripheral and central neuronal sensitization or hyperactivity)^32^ that ensues after the acute to chronic pain transition may be initiated and maintained, in part by peripheral mechanisms.^33^^,^^34^ This is most evident in the clinic when local anesthetic nerve blocks innervating painful regions transiently relieve acute and chronic pain of varying etiologies.^35^^,^^36^^,^^37^ Several sites in the PNS are believed to be pain generators important in the maintenance of chronic pain.^38^^,^^39^^,^^40^ These include specialized nerve fibers in the skin and deeper tissues, axon terminals, the sites of injured nerves, and dorsal root ganglia (DRG).^41^^,^^42^^,^^43^^,^^44^^,^^45^^,^^46^ This concept is fundamentally important in guiding future chronic pain therapeutics development.^11^^,^^47^^,^^48^ The advantages of targeting peripheral sites such as primary afferent neurons (specialized pain-sensing neurons), or satellite cells in DRG or elsewhere may avoid adverse events often observed clinically with current chronic pain medications related to systemic exposure. Additional benefits of treating locally accrue due to selectively inhibiting pain initiation at the source while preserving desirable sensory inputs such as touch and proprioception.^40^^,^^49^^,^^50^^,^^51^ There is also the potential to reverse central sensitization^34^^,^^52^ when correcting localized peripheral imbalances in excitatory and inhibitory neuronal signaling. Localized delivery of therapeutics targeting the PNS creates new challenges and opportunities.

### The advantages of rdHSV-Based CA8∗ gene therapy for chronic pain management

rdHSV embodies significant benefits for delivering novel neuromodulation therapies for chronic pain management and the treatment of other neurological disorders. After infection, rdHSV undergoes efficient retrograde transport through axons to target neuronal nuclei. Once it reaches the neuronal nucleus, it persists in a latent state as a large double-stranded episomal element that is disease-free because it cannot integrate or replicate.^53^^,^^54^ Another major advantage of this rdHSV vector system is that it has the capacity to provide long-term transgene expression within the nucleus of targeted cells after a single treatment.^55^^,^^56^ rdHSV is particularly appealing as localized therapy because of the efficient transduction of PNS neurons (i.e., DRG) after injections of skin and other tissues. Limited biodistribution of rdHSV and a lack of immunogenicity represent other known advantages.^57^ By infecting only the region of the body that is impacted by the neurological disease, it minimizes unwanted exposure to unaffected regions of the body, minimizing the possibility of side effects and maximizing therapeutic efficacy. rdHSV-CA8∗ is a highly defective vector incorporating a variety of genomic deletions including essential immediate-early (IE) genes.^58^ Deleting these viral genes reduces the overall immunogenicity of these vectors and allows for large transgene inserts useful in treating a variety of neurological conditions and redosing of this type of therapy.^27^^,^^28^^,^^29^

### vHCA8∗WT analgesic targeting vector decreases neuronal excitability by activating neuronal Kv7 voltage-gated potassium channels

It is known that increased CA8∗ expression leads to decreased endoplasmic reticulum calcium release,^14^^,^^16^^,^^17^^,^^18^^,^^20^^,^^21^ resulting in lower cytosolic free calcium concentration and analgesia.^14^^,^^16^^,^^17^^,^^18^^,^^20^^,^^21^ KV7 voltage-gated potassium channels are the only known neuronal potassium channels activated by lower cytoplasmic free calcium to produce so-called M-currents (IM) through calmodulin-dependent and -independent mechanisms.^59^^,^^60^^,^^61^^,^^62^^,^^63^ IM slow currents regulate neuronal excitability and produce analgesia in part by prolonging neuronal after hyperpolarization (AHP), which restricts the firing of action potentials (APs) and the propagation of afferent nociceptive signals carrying pain.^64^^,^^65^ In contrast, apparently all other potassium channels that could be involved in mediating AHP, require higher calcium for activation.^15^^,^^59^^,^^61^^,^^62^^,^^63^^,^^64^^,^^65^^,^^66^ Neuronal somata infected with vHCA8WT and controls did not differ in size, RMP, AP peak amplitude, or AP duration. However, neuronal somata infected with vHCA8WT exhibited much larger AHP.^15^ Specifically, vHCA8WT AHPs, when compared with controls, exhibited significantly larger AHP peak amplitude with −11.65 ± 3.25 vs. −6.72 ± 2.5 mV (*p* < 0.01), longer AHP duration 321.77 ± 170.45 vs. 134.24 ± 72 ms (*p* < 0.05) and longer AHP duration at 50% amplitude 91.93 ± 51.39 vs. 36.63 ± 20.96 ms (*p* < 0.05), respectively.^15^ Because prolonged AHP results in decreased neuronal excitability, firing, and reduced nociceptive traffic to primary afferent synaptic terminals, these findings are consistent with vHCA8∗ activating Kv7 voltage-gated potassium channels leading to decreased neuronal excitability resulting in vHCA8WT-driven antinociception and analgesia.^14^^,^^15^ While small molecule oral Kv7 activators have been translated from bench to bedside to treat epilepsy and chronic pain, there are no selective Kv7 activators currently available for clinical use. Off-target effects and serious toxicity led to the removal of these therapeutics from the market.^67^^,^^68^

Thus, vHCA8∗ gene therapy, which seems to result in downstream activation of Kv7 and decreased primary afferent excitability, acts as a biological neuromodulator that may provide a long-lasting alternative to opioids for clinical analgesia and anti-hyperalgesia. Gene therapy provides another potential advantage of localized delivery of this novel Kv7 activator, avoiding off-target effects and other safety concerns seen with the systemic administration of prior Kv7 activators used to treat epilepsy and various forms of chronic pain successfully.^67^^,^^68^

### Potential therapeutic implications of these studies

Several important implications can be drawn from these non-GLP safety data in mice. These results suggest vHCA8∗ gene therapy in naive mice provides sustained analgesic effects for ≤6 months, comparable with HD morphine administration (>100 mg/day in an average adult). This represents a significant advance in pain management, offering a long-duration solution with a single treatment. The translation of murine analgesic responses to human morphine equivalents suggests vHCA8∗ could represent a potent alternative to long-term opioid therapy. Therefore, patients requiring chronic pain management might rely less on opioids, reducing risks of dependency, abuse, and adverse effects associated with HD opioid use. These results also provide for mechanistic insights and validation of this novel Kv7 activator. The use of both mechanical and thermal withdrawal assays (von Frey and Hargreaves methods) validates the analgesic effects across different nociceptive pathways without loss of motor functions (weight bearing) or observations in behavior. This broader sensory applicability supports the robust efficacy of vHCA8∗ gene therapy. Allometric conversion provides a framework to predict human-equivalent morphine doses, enhancing the clinical relevance of these findings assisting with translation to the clinic. The estimated >100 mg/day of oral morphine equivalence demonstrates that this gene therapy may significantly impact clinical approaches to pain, particularly for conditions resistant to conventional pharmacotherapies. A single vHCA8∗ treatment potentially provides significant advantages in that it appears capable of sustained profound analgesic effects, offering a convenient therapeutic option compared with repetitive daily systemic drug dosing. This approach has the potential to minimize healthcare resource utilization by reducing the need for frequent interventions and monitoring. Although these results are promising, further research is required to confirm these initial safety data and support manufacturing scalability of vHCA8∗ therapy for human trials and eventual market approval.

### Translational and regulatory considerations in chronic pain analgesic development

Despite promising advancements, significant barriers remain to bringing an array of novel chronic pain therapeutics so-called analgesics to the market.^69^^,^^70^^,^^71^ A failure to translate from animal models to humans in well-controlled human clinical trials has plagued the development of many analgesic candidates in development.^72^ While this has implicated the methods of measuring pain in animal models and species differences,^71^ it may also be relevant that these measurements in animal models have largely been focused on anti-hyperalgesia and not true analgesia in comparison with clinically useful positive analgesic controls. The NK1 receptor antagonist studies is a noteworthy example that demonstrated potent anti-hyperalgesia in animal models but failed to show significant improvement on baseline nociception (analgesia) in those models failed in human clinical studies.^72^ In our prior CA8∗ gene therapy studies and herein, we show significant dose-dependent analgesia in response to various painful stimuli in multiple models.^14^^,^^15^^,^^16^^,^^17^^,^^18^^,^^19^^,^^20^^,^^21^ In addition, selective activators of Kv7 channels are known to reverse mechanical allodynia and thermal hyperalgesia in rat models of inflammatory, neuropathic and bone cancer pain, and have been translated successfully to human pain treatment.^68^^,^^73^^,^^74^^,^^75^^,^^76^^,^^77^^,^^78^^,^^79^ Further, these medications were shown in clinical practice and small-scale controlled human clinical studies to produce analgesia comparable to opioids, but without dependence, tolerance, and opioid-like complications.^80^ Unfortunately, these selective Kv7 activators, despite potentially offering highly effective long-term analgesia, were removed from the market due to serious irreversible toxicity. While acetaminophen and some popular non-steroidal anti-inflammatory drugs (e.g., diclofenac, meclofenamate, and celecoxib) are available effective chronic pain therapies,^81^^,^^82^^,^^83^^,^^84^ they seem to be less selective in their mechanism of action, and likely weaker Kv7 activating compounds with serious side effects when used long-term.^84^^,^^85^^,^^86^^,^^87^ There remains an unmet need for long-acting Kv7 activating medications in chronic pain management.

Nonetheless, vHCA8∗ gene therapy faces stringent regulatory requirements, and drug developers must navigate these efficiently to bring therapies to clinical use. However, new treatments for chronic pain due to knee OA may benefit from an established regulatory pathway for treating this condition. The knee OA pain population is well defined, and regulatory bodies have approved numerous pain therapeutics with well-characterized end points. Additionally, the U.S. Food and Drug Administration provides guidance documents to industry related to drug development for OA (Osteoarthritis: Structural Endpoints for the Development of Drugs).

## Materials and methods

### Bioengineering, manufacturing, and quality control of rdHSV-based CA8∗ analgesic primary afferent targeting vector

The HSV gene therapy used in our current study is based on JDNI8, which are highly defective non-replicating viral vectors that are deleted for functional expression of all the viral IE genes.^27^^,^^28^^,^^29^ These rdHSV vectors provide an efficient delivery system to the PNS that selectively establishes natural lifelong latency within targeted neurons after retrograde transport of viral particles to the DRG nerve cell body. Together these viral modifications provide a non-cytotoxic disease-free vector capable of long-term episomal maintenance in sensory neuron somata with >6 months of continued, robust transgene expression.^28^ Deleting all IE genes critical to viral replication, making these vectors disease free and less immunogenic than most alternative gene therapies.^15^

A diagram of our rdHSV constructs is shown in Figure S3.^15^ These vectors have been used previously in proof-of-concept studies of chronic pain.^14^ We set out to bioengineer, manufacture, quality control (QC), and test two vectors (1) rdHSV-*CA8∗WT-T2A-GFP* (vHCA8∗WT) and (2) rdHSV-*CA8∗MT-T2A-GFP* (vHCA8∗MT) negative control (containing the S100P null point mutation^88^) in the JDNI8 rdHSV backbone.^15^ The vHCA8∗MT incorporates the S100P mutation into the CA8∗ targeting vector representing most rigorous negative control possible due to the nearly complete degradation of CA8 cellular protein associated with rapid proteasome-mediated destruction, but otherwise is identical to the vHCA8∗WT vector system.^88^ The *T2A* sequence causes ribosome skipping during translation, which allows multiple proteins to be expressed separately from a single mRNA downstream of a strong promoter.^89^ The result is distinct, functional proteins (CA8∗ and GFP) without requiring additional processing steps, or the use of separate promoter-regulatory constructs for driving therapeutic and reporter transgene expression. The transgenes were inserted downstream of the CAG^90^ promoter. We used this strong promoter system based on the cytomegalovirus IE enhancer element; the promoter region, first exon and the first intron of the chicken beta-Actin gene; and the splice acceptor of the rabbit beta-globin gene (CAG) that is known to be highly active in a broad array of cell types. These later generation nontoxic rdHSV vectors integrate transgene inserts at the *ICP4* locus site.^29^

The lab-scale process used to produce both vectors was described previously.^15^ Vectors were grown to high-titer using modified U2OS ICP4/ICP27-complementing (U2OS-4/27) cells at a multiplicity of infection of 0.00005 in VP-SFM MEDIA (Thermo Fisher Scientific) for 1 h at 37°C in a CO_2_ incubator. On ∼D8, the CFs displayed ∼90% CPE, and the next day, NaCl was added to 0.45 M, and the CFs rocked for 4 h. Virus supernatant was harvested and purified using a brief centrifugation step to remove cellular debris, a 0.8-micron CN filtration (Thermo Fisher Scientific) step followed by centrifugation at 43,000×*g* for 45–90 min, followed by dPBS wash and second identical centrifugation step. The vector was resuspended in dPBS with sterile glycerol added to a final volume of 10% and vialed in 10 μL (actual volume 12.5 μL) aliquots in cryovials and stored at −80°C. The retained biologic activity was determined by standard plaque assay on U2OS-4/27 complementing cells.^91^ After purification of vector stocks, QC testing was performed using a series of standard operating procedures. QC testing consisted of assessing the infectious viral titers (PFU/mL) by standard plaque assay, number of genome copies (gc/mL), level of host cell and vector dsDNA (PicoGreen, Thermo Fisher Scientific), host cell and vector protein contamination (NanoOrange, Thermo Fisher Scientific), and tests for mycoplasma and endotoxin contamination. Additionally, sterility was tested using aerobic and anaerobic conditions on various media. Stocks that passed QC/QA criteria (see Table S1) were subsequently tested in further studies at University of Miami, Miller School of Medicine. Quality assurance/QC was performed and product released according to the methods and criteria listed in Tables S1 and S2. To verify that these vectors produce the proper-sized proprietary CA8 protein (**∗CA8**) in cultured DRG neurons, western blots were run with DRG cell lysates, which were harvested after D2 infection and probed with CA8∗ antibodies. The vHCA8∗WT vectors detected high levels of CA8∗ (Figure S3), while vHCA8∗MT vector expressed greatly reduced CA8∗ protein levels in comparison to the β-actin loading control (data not shown). These results were similar to AAV-*CA8∗WT* and *MT* vectors produced and reported previously.^21^ These highly purified, high-titer rdHSV constructs that passed release specifications (Tables S1 and S2) were then used in the reported safety, efficacy, biodistribution, and shedding studies.

### HSV virus delivery

We used naive 8- to 10-week-old male BALB/c mice anesthetized for painful procedures by i.p. injection of ketamine, xylazine and acepromazine. vHCA8∗WT or vHCA8∗MT viral particles (6 μL or 1E6 PFU HD, 1E5 PFU MD or 1E4 PFU low dose) were injected into the KJ using a 50 μL microsyringe with a 30G needle.

### Pain behavior analyses

Animals were habituated to the testing environment daily for at least four consecutive days before experimental testing and Baseline establishment. The room temperature and humidity remained stable for all experiments. Mechanical responses using Dixon’s up-down method with calibrated von Frey filaments,^92^ thermal responses using Hargreave’s method,^30^ and motor functions using weight-bearing, were performed essentially as described elsewhere.^14^ Briefly, for testing mechanical sensitivity, animals were put under an inverted round plastic box (radius, 9 cm; height, 11 cm) on an elevated metal mesh floor and allowed 60 min for habituation before the threshold test. The plantar surface of each hind paw was stimulated with a series of calibrated von Frey fibers (from 0.4 to 6 g) (Stoelting Co.). The threshold was determined as the lowest force that evoked a consistent brisk withdrawal response.^20^^,^^93^ The test was performed on days as described in the figure legends. The Dixon up-down method was used to calculate 50% mechanical withdrawal thresholds.^20^^,^^92^ Thermal withdrawal latencies were performed in groups using the Hargreave’s method (Ugo Basile Thermal Plantar, Stoelting Co.).^14^ At no time did any treatment group reach the maximum thermal or mechanical withdrawal response. Weight-bearing distribution between the left (virus) and right (contralateral) hind limbs were tested by the Librae Incapacitance Tester (Ugo Basile) in mice after various treatments. Data are presented as the percentage of weight distributed on the left hindlimb calculated by the formula: [weight on the left hindlimb/(weight on the left + weight on the right)] x 100. Measurements were performed in naive BALB/c mice as described elsewhere.^14^ All testing was performed at about the same time of day by a trained investigator masked to treatment groups. There were *N* = 10 mice per group, unless noted otherwise. All procedures related to animal use and care were preapproved by the University of Miami Institutional Animal Use and Care Committee (IACUC).

### Biodistribution and shedding

Briefly, total genomic DNA was extracted from murine tissues collected after completion of the in-life testing experiments. Thermo Fisher Scientific TaqMan Fast Advanced Master Mix (Cat#: 4444557) was used to run *gD* qPCR with the Applied Biosystem QS3, and the JDNI8-*gD-CA8∗* vector was used to make the standard for measuring vHCA8∗ viral copies.^29^

The primers and probe used were as follows:

Forward primer: 5′-*CCCCGCTGGAACTACTATGACA*-3′,

Reverse primer: 5′-*GCATCAGGAACCCCAGGTT-*3′

Probe: 5′-FAM-*TTCAGCGCCGTCAGCGAGGA*-TAMRA-3′

### Statistical methods

Statistical methods were performed essentially as described elsewhere.^14^ The sample size was as noted in each figure legend. IBM SPSS Statistics 26 was used to directly calculate the significance between groups at each time point incorporating a Fisher’s LSD test. The number of mice used per assay group was based on power analyses using the observed variation in prior AAV8-CA8 assays.^20^ We estimated that *N* = 8–10 animals per group would provide >95% power at *p* = 0.05. No animals were excluded from our analyses.

## Data availability

Data that support the findings of this study are available after reasonable request from corresponding author.

## Acknowledgments

This work was funded in part from the 10.13039/100000065NINDS UH3 NS123964 (R.C.L.); 10.13039/100000065NINDS R21 NS105880 (R.C.L.); 10.13039/100000065NINDS 3R21NS105880-01S1 (R.C.L.); and 10.13039/100000005DOD W81XWH-19-1-0525 (R.C.L.). We thank the Department of Anesthesiology, Perioperative Medicine, and Pain Management, 10.13039/100006686University of Miami 10.13039/100008530Miller School of Medicine, Miami, Florida for additional support.

## Author contributions

G.Z.Z. contributed to the general study design, troubleshooting and experimental results relating to animal studies, data analyses, tables and figures and general manuscript preparation; W.F.G. contributed to the design, bioengineering, construction, production, troubleshooting and experimental characterization of HSV vectors, data generation and analyses, tables and figures and general manuscript preparation; M.B.K. contributed to the experimental results, and general manuscript preparation; J.C.G. contributed to the HSV vector design, drug manufacturing, troubleshooting, and general manuscript preparation; K.D.S. contributed to the overall design, manuscript preparation, and editing; R.C.L. contributed to the overall design of all studies, troubleshooting, data analyses, and manuscript preparation, editing, and submission.

## Declaration of interests

R.C.L. is inventor of PCT (61/847,405) and J.C.G. inventor of PCT (62/532,182). R.C.L., G.Z.Z., W.F.G., and J.C.G. are equity shareholders; R.C.L. and J.C.G. serve as management of Adolore Biotherapeutics, Inc. focused on commercialization of rdHSV-CA8 biotherapeutic candidates.

## Supplemental information

Document S1. Figures S1–S3, Tables S1 and S2

Document S2. Article plus supplemental information
