# Supplementary material for: Biosafety and efficacy of Kv7 activating rdHSV-CA8∗ analgesic gene therapy for chronic pain via the intra-articular route in mice
Source: Mol Ther. 2025 May 28;33(9):4447–58. doi: 10.1016/j.ymthe.2025.05.029 (PMC12432987; doi:10.1016/j.ymthe.2025.05.029)
Supplement: Supplementary file 1 [file mmc1.pdf]

YMTHE, Volume *33*

Supplemental Information

Biosafety and efficacy of Kv7 activating rdHSV-CA8* analgesic gene therapy for chronic pain via the intra-articular route in mice

Roy C. Levitt, Munal B. Kandel, Gerald Z. Zhuang, William F. Goins, Konstantinos D. Sarantopoulos, and Joseph C. Glorioso

SUPPLEMENTAL MATERIALS

TABLE S1. Summary of Non-GMP QC Release Methods and Acceptable Release Standards.

Aliquots of 10μl were used for QA/QC testing, and expression/toxicity in primary rat DRGs.

Test Method	Acceptable Range	Value	P/F
Viability: Titer on U2OS-4/27 complementing cells (PFU/mL)	> 1.0×10^8 PFU/mL	1.4×10^8 PFU/mL	-
Endotoxin (E.U./mL)	< 2.0 EU/mL	0.242 EU/mL	Pass
Sterility on chocolate blood agar grown aerobically out to 14-d	No growth	No growth	Pass
Sterility on chocolate blood agar grown anaerobically out to 14-d	No growth	No growth	Pass
Sterility on Sabouraud's agar grown aerobically out to 14-d	No growth	No growth	Pass
Sterility on Sabouraud's agar grown anaerobically out to 14-d	No growth	No growth	Pass
Sterility on BHF agar grown aerobically out to 14-d	No growth	No growth	Pass
Sterility on BHF agar grown anaerobically out to 14-d	No growth	No growth	Pass
Total DNA by PicoGreen	< 10 μg/mL	7.14 μg/mL	Pass
Total Protein by NanoOrange	< 20 μg/mL	12.4 μg/mL	Pass
Viral genome copies by qPCR (Genome Copies/PFU)	< 10,000 GC/PFU	4760 GC/PFU	Pass
Mycoplasma by PCR	No band on gel	No band on gel	Pass
Appearance	Clear-Milky no particulate	Clear-Milky no particulate	Pass
pH	6.6 – 7.0	6.6	Pass
Identity of V5-CA8 by western blot using V5 Ab	Positive	Positive	Pass

TABLE S2. Assessing Cellular Toxicity. MTT assay was used to assess cellular toxicity for vHCA8*WT and vHCA8*MT (these data are not show) vectors, using A. primary rat DRG, or B. mock infected DRG as a control.

	Test Method	Acceptable Range	Value	P/F
A	Toxicity/Cell Viability (MTT Assay) vs Control Vector in 1° DRGs	Ratio > 1.0	1.43	Pass
B	Toxicity/Cell Viability (MTT Assay) vs Mock-infected in 1° DRGs	Ratio > 0.8	1.11	Pass

FIGURE S1

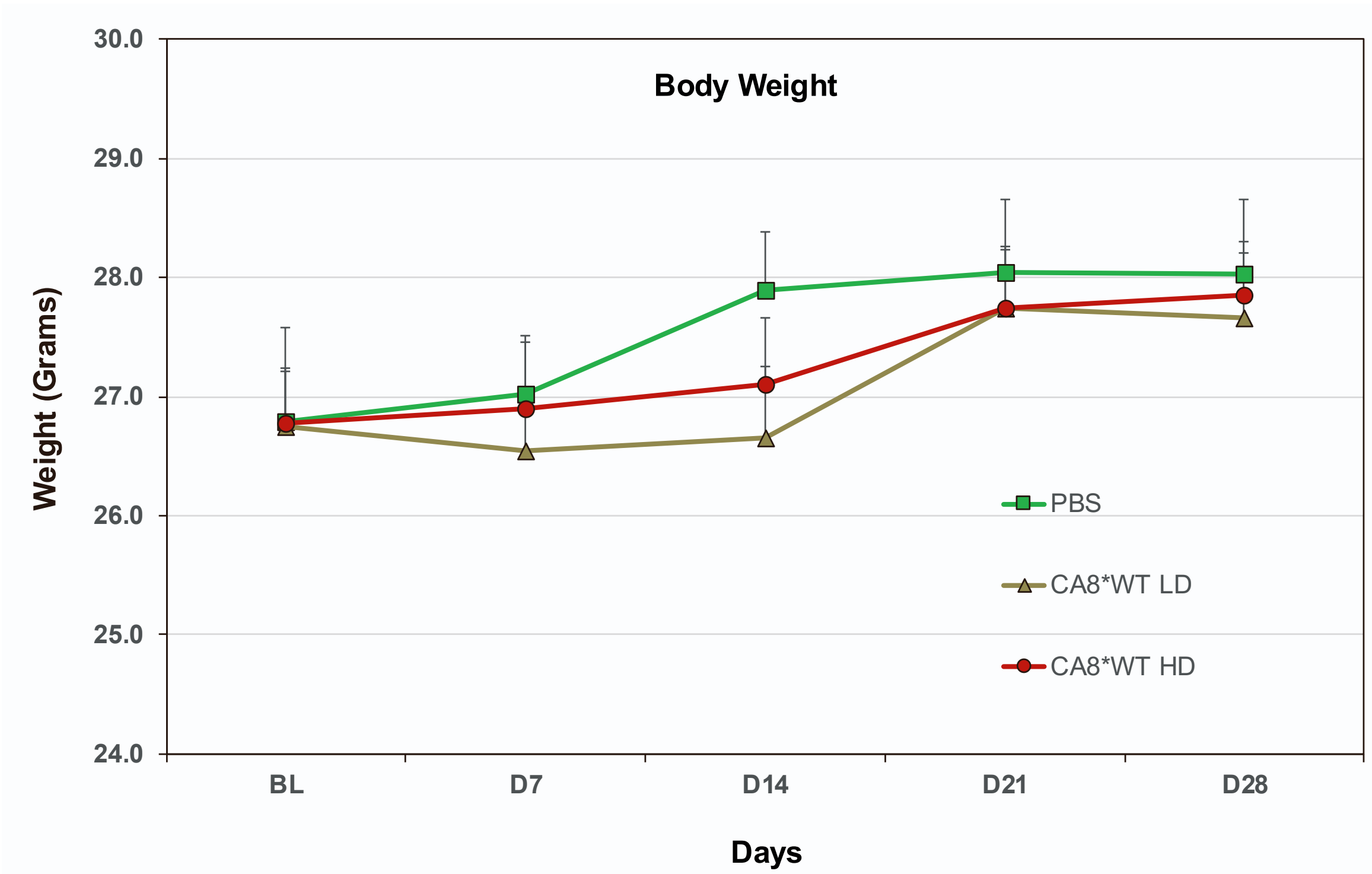

FIGURE S1. Body Weight (BW) Measurements in Naïve BALB/c Mice Treated with IA KJ Injections of rdHSV-CA8* Included in Safety, Efficacy, Biodistribution and Shedding Studies. BALB/c mice were injected with vHCA8* in the knee joint with vHCA8*WT high-dose (1E6 PFU), vHCA8*WT mid-dose (1E5 PFU), or PBS (vehicle)(negative control) in 6 μL volume, N=10 per group and animals were assigned to groups randomly. **Assessment of BW**. BW (grams) was measured daily for four days about the same time of day for each animal to establish a Baseline and then once weekly for 4 weeks. There were no significant variations in BW between groups for these mice through D28 using one-way ANOVA post hoc test. There was no significant differences between groups by repeated two-way ANOVA analyses [F(2,27)=0.25, P=0.78], or interactions between time points and groups [F(8,108)=0.68, P=0.71]. There was a significant difference found between timepoints for BW [F(4,108)=10.24, P=4.54E-07].

FIGURE S2

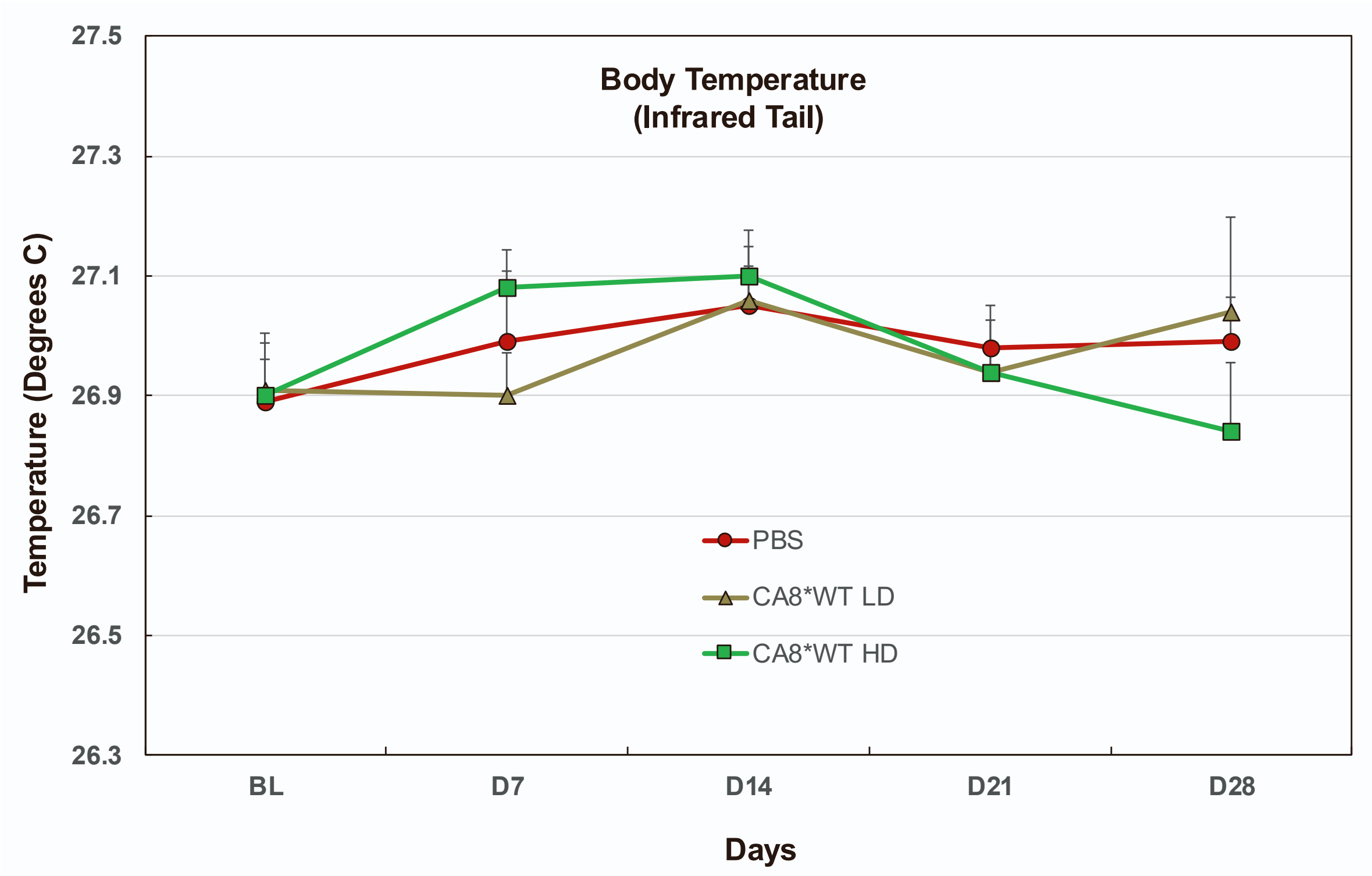

FIGURE S2. Body Temperature (BT) Measurements in Naïve BALB/c Mice Treated with IA KJ Injections of rdHSV-CA8* Included in Safety, Efficacy, Biodistribution and Shedding Studies. BALB/c mice were injected with vHCA8* in the knee joint with vHCA8*WT high-dose (1E6 PFU), vHCA8*WT mid-dose (1E5 PFU), or PBS (vehicle)(negative control) in 6 μL volume, N=10 per group and animals were assigned to groups randomly. **Assessment of BT.** BT was measured by an infrared thermometer (degrees C) on the tail surface nearest the body according to the manufacturer's instruction (BioSEB, BIO-IRB153, France) at about the same time of day for each animal. BT was measured daily for four days to establish a Baseline and then once weekly for 4 weeks. There were no significant variations in BT, as shown on various days using one-way ANOVA post hoc test. Two-way ANOVA analyses showed there were no significant differences between groups [$F(2,27)=0.019$, $P=0.98$]; or interactions between time points and groups [$F(8,108)=0.59$, $P=0.79$]. There was no significant differences in BT observed across timepoints [$F(4,8)=1.37$, $P=0.25$], $P<0.05$ was considered significant.

FIGURE S3

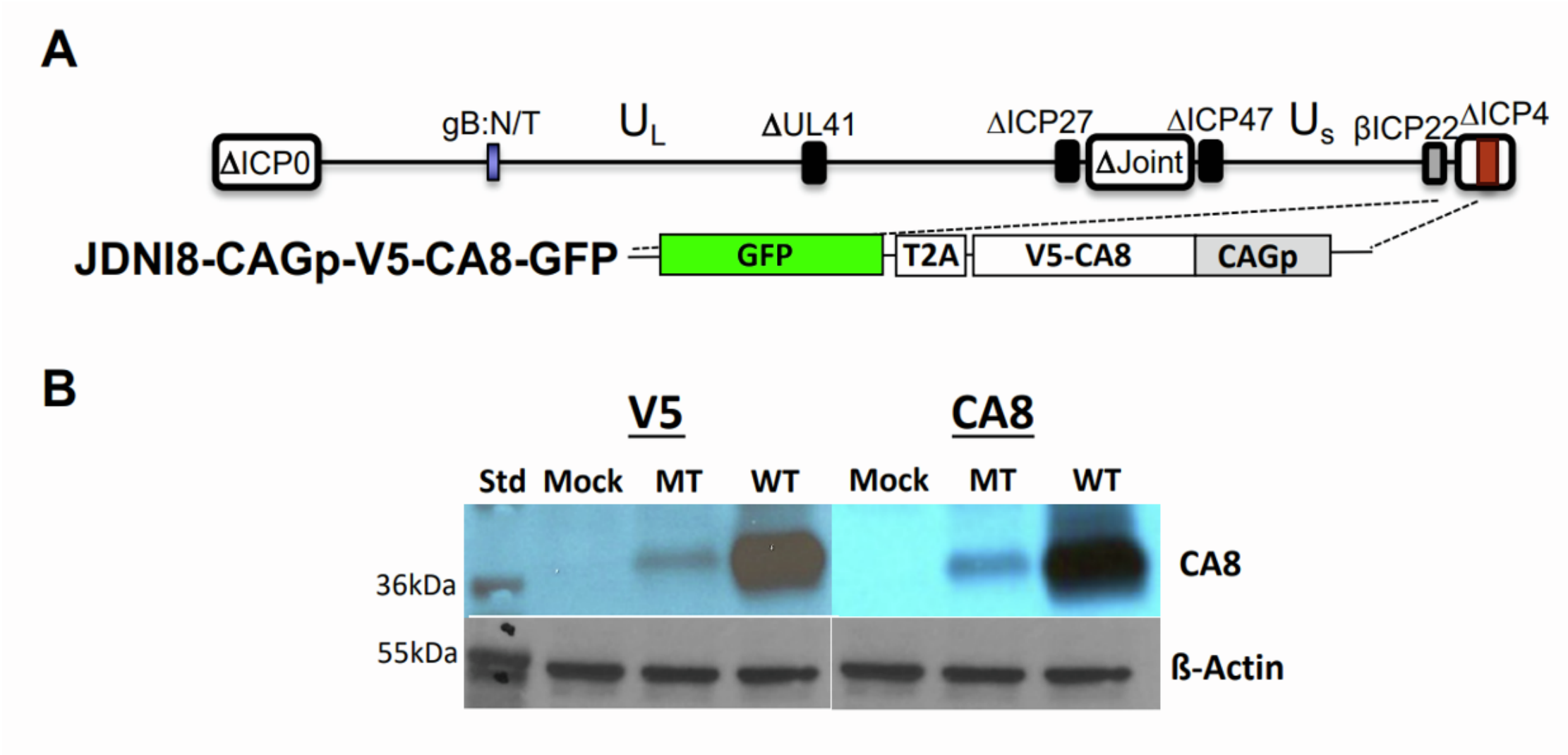

FIGURE S3. Schematic Diagram of rdHSV-CA8* (vHCA8*) Neuronal Targeting Vector Schematic and Constructs and Expression in Primary Rat DRG Neurons *In Vitro*. Panel A. The JDNI8 vector was derived from JDNI5 [1, 2]. The BAC elements are located between *lox*P sites in the UL37-UL38 intergenic region [3]. U_L, unique long segment; Us, unique short segment. Deletions in JDNI8 are indicated by black boxes. JDNI8 contains the entry-enhancing D285N/A549T(N/T) mutations in the gB gene (gB: NT) [4], and a CAG (CAGp)-CA8*-V5-T2A-eGFP cassette (green) in the ICP4 site (red). The blue rectangle box (βICP22) indicates the conversion of the ICP22 IE gene to early-expression kinetics by deletion of promoter TAATGARAT. **Panel B.** Western blots were processed for SDS-PAGE, transferred to PVDF membranes, and immunoblotted with antibodies to CA8 and the V5 tag using ß-actin as a loading control for primary rat DRG neuronal cell lysates 2 days after infection with vHCA8WT or vHCA8MT (MOI=5). The correct-sized band was identified in each case. vHCA8MTvirus was barely detectable above the background as compared to vHCA8WT virus. [From Kandel et al., 2024][5]

Supplemental References:

1. Miyagawa Y, Marino P, Verlengia G, Uchida H, Goins WF, Yokota S, Geller DA, Yoshida O, Mester J, Cohen JB, Glorioso JC. Herpes simplex viral-vector design for efficient transduction of nonneuronal cells without cytotoxicity. Proc Natl Acad Sci U S A. 2015;112(13):E1632-41. Epub 20150316. doi: 10.1073/pnas.1423556112. PubMed PMID: 25775541; PMCID: PMC4386379.
2. Miyagawa Y, Verlengia G, Reinhart B, Han F, Uchida H, Zucchini S, Goins WF, Simonato M, Cohen JB, Glorioso JC. Deletion of the Virion Host Shut-off Gene Enhances Neuronal-Selective Transgene Expression from an HSV Vector Lacking Functional IE Genes. Mol Ther Methods Clin Dev. 2017;6:79-90. Epub 20170616. doi: 10.1016/j.omtm.2017.06.001. PubMed PMID: 28702475; PMCID: PMC5493822.
3. Gierasch WW, Zimmerman DL, Ward SL, Vanheyningen TK, Romine JD, Leib DA. Construction and characterization of bacterial artificial chromosomes containing HSV-1 strains 17 and KOS. J Virol Methods. 2006;135(2):197-206. Epub 20060502. doi: 10.1016/j.jviromet.2006.03.014. PubMed PMID: 16647145.
4. Uchida H, Chan J, Goins WF, Grandi P, Kumagai I, Cohen JB, Glorioso JC. A double mutation in glycoprotein gB compensates for ineffective gD-dependent initiation of herpes simplex virus type 1 infection. J Virol. 2010;84(23):12200-9. Epub 20100922. doi: 10.1128/JVI.01633-10. PubMed PMID: 20861246; PMCID: PMC2976401.
5. Kandel MB, Zhuang GZ, Goins WF, Marzulli M, Zhang M, Glorioso JC, Kang Y, Levitt AE, Kwok WM, Levitt RC, Sarantopoulos KD. rdHSV-CA8 non-opioid analgesic gene therapy decreases somatosensory neuronal excitability by activating Kv7 voltage-gated potassium channels. Front Mol Neurosci. 2024;17:1398839. Epub 20240509. doi: 10.3389/fnmol.2024.1398839. PubMed PMID: 38783904; PMCID: PMC11112096.
